# Comparison of Two Coronary Anastomosis Techniques in Terms of Flow Rate in Porcine Hearts

**DOI:** 10.21470/1678-9741-2024-0073

**Published:** 2025-02-05

**Authors:** Safa Gode, Mucahit Polat, Elif Guneysu, Timucin Aksu, Olgar Bayserke, Muhammed Bayram, Ulku Kafa Kulacoglu, Taner Iyigun, Zihni Mert Duman, Oznur Inan

**Affiliations:** 1 Department of Cardiovascular Surgery, Istanbul Mehmet Akif Ersoy Thoracic and Cardiovascular Surgery Training and Research Hospital, Istanbul, Turkey; 2 Department of Cardiovascular Surgery, Elazig Fethi Sekin City Hospital, Elazig, Turkey; 3 Istanbul Experimental Research Development and Education Center, Istanbul Mehmet Akif Ersoy Research Development and Training Center, Istanbul, Turkey

**Keywords:** Coronary Anastomosis Technique, Hemodynamic Performance, Graft Flow, Coronary Artery Bypass, Graft Patency

## Abstract

**Introduction:**

The quality of coronary anastomoses is one of the important parameters that may affect graft patency in coronary artery bypass grafting patients. Therefore, we compared two different anastomotic techniques to improve graft flow and patency rates.

**Methods:**

This study was conducted by performing two different fashions of anastomosis with a human saphenous vein graft on 24 various coronary segments of five postmortem porcine hearts. Each arteriotomy was used for both anastomotic techniques. In the first method, epicardial fat tissue around the coronary artery was involved to the saphenous vein anastomosis line (coronary wall and epicardial fat tissue [CWE] technique). In the second method, the saphenous vein graft was sutured to the coronary wall only, without involving epicardial fat tissue (only coronary wall [OCW] technique).The time it tookfor 30 cc of 0.9% isotonic saline solution to pass through the anastomosis in a free-flow fashion by gravity was measured following each technique. Additionally, the anastomotic areas in mm^2^ were measured and compared between the two techniques.

**Results:**

The mean flow time for the CWE technique was 77.5 ± 21.4 seconds, whereas for the OCW technique, it was 87.2 ± 19.5 seconds (*P*<0.001). The flow rates were 23.2 ml/min and 20.6 ml/min, respectively. The anastomotic area was 3.947 mm^2^ for the CWE technique and 1.430 mm^2^ for the OCW technique.

**Conclusion:**

When the sutures penetrate both the epicardial fat tissue and the coronary artery wall simultaneously, a larger anastomosis area can be created. Consequently, potentially better graft flow and hemodynamic performance could be achieved.

## INTRODUCTION

Intraoperative graft flow measurement has been recommended by the Guidelines for Myocardial Revascularization of the European Association for Cardio-Thoracic Surgery and the European Society of Cardiology for coronary artery bypass grafting (CABG) for more than a decade. The guidelines advocate the use of intraoperative graft flow assessment, such as transit-time flow measurement (TTFM), to ensure the quality of the bypass grafts and reduce the risk of perioperative myocardial infarction and long-term graft failure (Class I recommendation, Level of Evidence C)^[1]^. There are several factors that can affect TTFM of grafts during CABG. Some of these factors include graft size, coronary resistance, graft kinking, dissection, or spasm, competitive flow, and anastomotic failure^[2]^. The quality of coronary anastomoses is one of the most important factors in determining the patency of grafts and the clinical outcomes of patients after CABG. Studies have shown that anastomotic failure is a primary cause of early graft failure, and the quality of the anastomosis is a strong predictor of graft patency and long-term clinical outcomes^[3,4]^. Therefore, it is crucial for surgeons to prioritize the quality of the anastomosis during CABG.

There have been several studies investigating the impact of different coronary anastomosis techniques on graft patency and clinical outcomes. Tsukui et al.^[5]^ examined the relationship between anastomotic length and graft patency. They demonstrated that longer arteriotomies resulted in a larger anastomotic area and smaller angles between the coronary artery and saphenous vein graft, potentially leading to smoother graft curvatures and improved hemodynamic performance. Therefore, they found that longer anastomoses resulted in better graft patency and reduced rates of perioperative complications compared to shorter anastomoses.

In another study, the impact of different anastomotic techniques, suture materials, suture techniques, and adjunctive devices on graft patency and clinical outcomes was investigated. Therefore, it has been shown that the use of running sutures, rather than interrupted sutures, can result in better graft patency and improved clinical outcomes. Additionally, the use of adjunctive devices such as stabilizers has been found to enhance the accuracy and quality of the anastomosis^[6]^.

Despite all these studies in the literature, the definitive optimal surgical technique for coronary anastomoses has not yet been determined. In this study, we aimed to compare two coronary anastomosis techniques in terms of anastomotic area and flow rate.

## METHODS

**Fig. 1:**
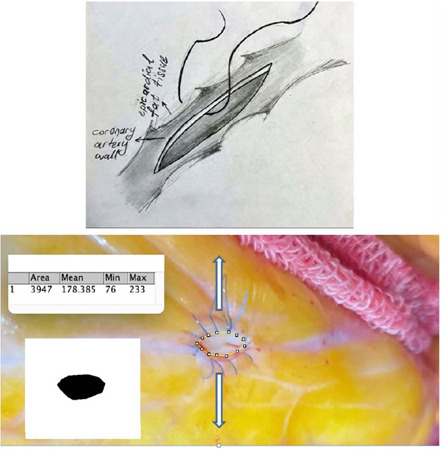
The onostomoses performed with the coronary wall and epicardial fat tissue technique

**Fig. 2:**
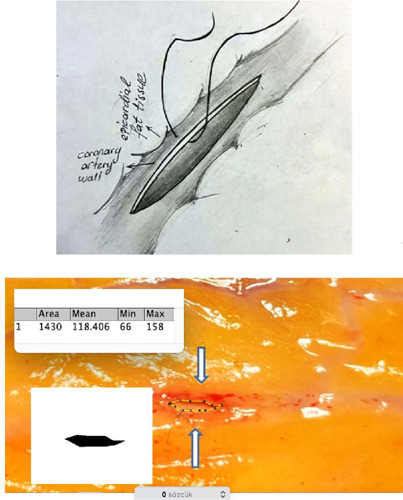
The anastomoses performed with the only coronary wall technique.

This study was conducted using isolated postmortem porcine hearts, with a total of five hearts utilized to create 24 anastomoses. These anastomoses were performed on 13 different segments of the left anterior descending arteries, seven segments of the posterior descending arteries, and four segments of the circumflex arteries on all hearts. Qualified human saphenous vein graft with 15 cm length remaining from a male patient who underwent CABG were used for these anastomoses. All anastomoses were performed by the same surgeon using 8/0 double round needle Prolene® and a running suture, aiming to minimize variability in the surgical technique and reduce potential confounding factors that may impact the study results. Two different techniques were applied for anastomoses. Initially, anastomoses were performed by penetrating the suture through the coronary artery wall and epicardial fat tissue together (Figure 1). The group that used this technique was defined as coronary wall and epicardial fat tissue (CWE) group (n=24). In the alternative technique, the suture was penetrated only through the coronary artery wall, excluding the epicardial fat tissue, in the traditional manner (Figure 2). This group was designated as the only coronary wall (OCW) group (n=24). In both techniques, sutures were consistently applied with equal depth, ensuring they did not penetrate too deeply into the coronary wall.

### Ethical Issues

The National Experimental Animals Ethics Committee approved the study with numbered IMAEH 54374/2023. Study was also performed according to the Guide for the Care and Use of Laboratory Animals published by the National Institute of Health.

### Measurement Technique

**Fig. 3:**
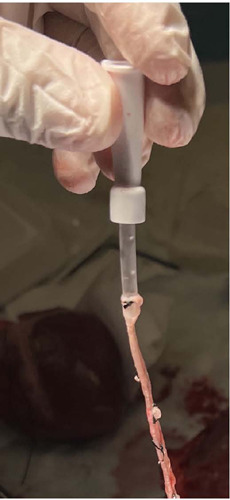
Saphenous vein graft cannulated with 22 French nasogastric catheter to help to minimize resistance and ensure a smooth and consistent flow of fluid during the measurement.

**Fig. 4:**
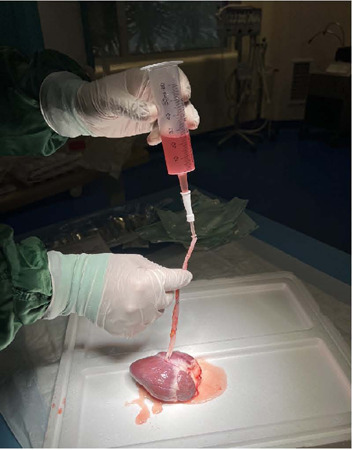
Measurement setup: the gavage syringe which stopper was removed and filled with 50 cc of saline, connected to saphenous vein graft via nasogastric catheter, and held vertically as distance from anastomosis to gavage syringe adjusts as 12 cm.

The proximal end of the saphenous vein graft was cannulated using a 22 French nasogastric catheter to minimize resistance and ensure a smooth, consistent flow of fluid during measurement. The nasogastric catheter was then connected to a 50 ml gavage syringe (Figure 3). The distal end of the saphenous graft was anastomosed, and the stopper from the gavage syringe was removed and filled with 50 cc of isotonic saline solution (0.9%). It was then connected to the saphenous vein graft via the nasogastric catheter and held vertically, adjusting the distance from the anastomosis to the gavage syringe to 15 cm (Figure 4).

The free flow rate is directly proportional to flow time. In other words, the time it takes for a certain amount of isotonic saline solution to pass through the anastomosis is directly proportional to the amount that passes in the certain time. Based on this, the passage time of 30 cc of isotonic saline solution through the anastomosis was directly measured, and the flow rate of the solution passing through the anastomosis in 60 seconds was indirectly calculated.

We explained the reason for using the indirect measurement method as follows: during the measurement, when the fluid level decreases, the pressure of the fluid decreases at the bottom of the syringe. Therefore, equal fluid pressure should have been provided for all measurements. The time it takes for the fluid in the syringe to decrease from 50 cc to 20 cc through free flow by gravity after declamping the saphenous vein graft was calculated. Thus, an equal flow pressure was maintained during all measurement.

A single saphenous vein graft, measuring 15 cm in length, was utilized for all anastomoses. This approach was chosen to prevent potential variations in size and overall graft quality among different grafts. After completing the measurements for the first technique, the anastomosis was carefully dismantled without causing harm to the coronary artery wall, preparing for the next anastomosis. At this stage, utmost care was taken to avoid damaging the coronary wall, ensuring the integrity of the results. The second anastomosis technique was then applied to the same arteriotomy, using the same saphenous vein graft, and flow time measurements were performed again.

Both anastomosis techniques were applied to every arteriotomy on various segments of different coronary arteries of porcine hearts. This standardized the measurement of flow for both anastomosis techniques, ensuring consistency across all variables, including coronary artery diameter, anatomical variation, and proximal or distal stenotic segments, etc.

If the OCW technique was initially applied to the first arteriotomy, the CWE technique was applied first to the second arteriotomy. This order was consistently followed for all anastomoses, ensuring an equal number of opportunities for each technique on the same arteriotomy, thereby promoting accurate and reliable measurements. After using both techniques, the tip of the saphenous vein graft was minimally trimmed to ensure the graft’s continued health and viability for subsequent use. Furthermore, the comparison between the two techniques was also conducted without using a saphenous vein graft, focusing on the anastomotic area (Figure 1, Figure 2). The anastomotic area was measured using the Image J method, a Java-based image processing program developed at the National Institutes of Health and the Laboratory for Optical and Computational Instrumentation (University of Wisconsin)^[7,8]^.

### Statistical Analyses

Statistical analysis was performed with IBM Corp. Released 2013, IBM SPSS Statistics for Windows, version 22.0, Armonk, NY: IBM Corp. Whether distribution of continuous variables was normal was determined by Shapiro-Wilk test. Values displaying normal distribution were expressed as mean ± standard deviation. Statistical comparison of quantitative data was performed with paired sample *t*-test for continuous variables displaying normal distribution. Wilcoxon signed-rank test was used for continuous variables not displaying normal distribution. A value of *P*<0.05 was considered statistically significant.

## RESULTS

**Fig. 5:**
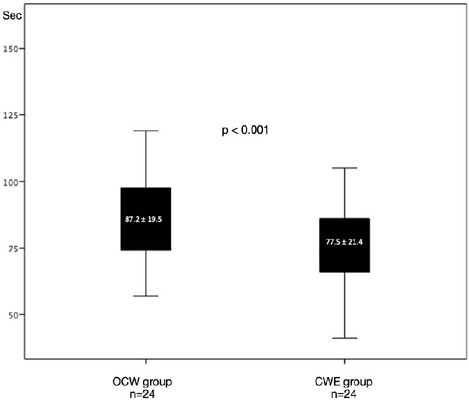
Graphic that shows statistical difference between the only coronary wall (OCW) group and the coronary wall and epicardial fat tissue (CWE) group in terms of flow rate.

We observed that the average time for 30 cc of isotonic saline solution to pass through the anastomoses was 77.5 ± 21.4 seconds in the CWE group and 87.2 ± 19.5 seconds in the OCW group (*P*<0.001) (Figure 5). This indicates that the same amount of isotonic saline solution passed through the anastomosis at a statistically significantly longer time in the OCW group. Additionally, when calculating the flow per minute, it was 23.2 ml/min in the CWE group and 20.6 ml/min in the OCW group. The results show a significantly higher flow in the CWE method compared to the OCW group.

The coronary anastomotic area measured using the ImageJ method was 3.947 mm^2^ for the CWE technique and 1.430 mm^2^ for the OCW technique. These findings suggest that the CWE method resulted in a larger anastomosis area compared to the OCW method (Figure 1, Figure 2).

One of the anastomoses failed due to the frailty of the coronary wall and was consequently excluded from the study.

## DISCUSSION

The quality of the coronary anastomosis is crucial for the success ofCABG. Poorly constructed anastomoses can lead to graft failure, which can result in myocardial ischemia, infarction, and even death. Therefore, it is essential to use the most effective and safe anastomosis techniques to ensure the best possible outcomes for CABG patients. There is no universally accepted standard technique for end-to-side or side-to-side coronary anastomosis, and the specific approach used can vary among surgeons. We conducted a study to compare between two types of coronary anastomosis techniques in postmortem porcine hearts, focusing on their effect on graft flow. Accordingly, in the CWE technique, the suture of the coronary anastomoses not only penetrates the coronary wall but also the large epicardial fat tissue together. To clarify, the suture in CWE technique is passed through both the coronary wall and the epicardial fat tissue, resulting in a thicker anastomosis line compared to the OCW technique. In the OCW technique, the suture is only passed through the coronary wall and not through the epicardial fat tissue. This creates a thinner anastomosis line compared to the CWE technique.

We observed that the CWE technique resulted in a larger anastomosis area, which we attributed to the traction applied to the sidewalls of the arteriotomy in a sideways direction. Based on the findings of the study, it appears that the CWE technique may indeed improve hemodynamic parameters relative to the OCW technique. We investigated this hypothesis by applying both anastomosis techniques to the same arteriotomy one after another. We found that when 30 ml of isotonic saline solution was passed through the anastomoses performed using the CWE technique, it took 77.5 ± 21.4 seconds, whereas the same amount of isotonic saline solution took 87.2 ± 19.5 seconds to pass through the anastomoses performed using the OCW technique (*P*<0.001). Nevertheless, average flow was 23.2 ml/min in CWE group and 20.6 ml/min in OCW group. Cause the shorter the time for isotonic saline solution to pass through the anastomosis in the CWE group, the better the average flow in the CWE group. This could be thanks to the larger anastomosis area observed in the CWE technique, which may have allowed for better flow.

Numerous studies have explored graft flow in CABG patients, with most being clinical and utilizing flowmeters. Graft flow values can be influenced by various factors such as vasospasm, graft kinking, distal runoff, variable inflow pressure, and, importantly, the quality of anastomoses. To exclusively assess anastomoses, our animal model study aimed to eliminate the effects of these other parameters. Thus, we standardized all other variables to focus solely on the assessment of anastomoses.

Hiroyuki Tsukui et al.^[5]^ conducted a model study emphasizing the importance of anastomosis length in improving hemodynamics. They found that a longer arteriotomy provides a larger anastomosis area, resulting in a small graft angle and a smoother transition in graft curvature, contributing to laminar anastomotic flow and better hemodynamic performance. In our study, we adopted a different approach to achieve a larger anastomosis area, although we did not specifically investigate the angle of anastomoses and laminarity of flow. We believe that increasing the anastomosis area may lead to improved laminarity of flow. Tsukui et al.^[5]^ also suggested that a longer arteriotomy can help avoid the purse-string effect, and we propose that the CWE technique may offer advantages in this regard. In a study on ex vivo porcine hearts, geometric construction errors at coronary anastomoses were observed to reduce the area of the anastomotic orifice, highlighting the importance of anastomotic geometry^[9]^. Similarly, we measured and compared the anastomotic area for two techniques (Figure 1, Figure 2), resulting in 3.947 mm^2^ for the CWE technique and 1.430 mm^2^ for the OCW technique. The anastomotic area was significantly larger in the CWE technique. Frailty and weakness of coronary arteries, especially in diabetic patients, may result in anastomosis tearing and leakage. We estimate that this problem may be common in anastomoses with OCW technique. On the contrary, anastomoses with CWE may be safer in terms of hemostasis and tearing because the epicardial fat tissue was involved (included) in the anastomoses. As support for this hypothesis, we excluded an anastomosis with the OCW technique due to frailty and subsequent failure in our study.

While we did not investigate the pulsatility index and diastolic flow pattern, our study revealed an average flow of 23.2 ml/min in the CWE group and 20.6 ml/min in the OCW group. According to the literature, a mean graft flow < 10-15 ml/min and a pulsatility index > 5 indicate poor graft hemodynamics, necessitating a re-examination of the graft and the anastomosis. In our study, both methods demonstrated graft flows > 15 ml/min, indicating satisfactory graft flow according to the literature^[10,11]^.

Additionally, Sottiurai et al.^[12,13]^ observed a relationship between neointimal hyperplasia distribution and flow pattern at the distal end-to-side anastomosis. Neointimal hyperplasia tends to occur at zones with low or reversed velocities. Similarly, some studies have indicated that local flow disturbance may significantly contribute to neointimal hyperplasia development at vascular end-to-side anastomoses^[14,15]^. Considering these findings, it is plausible that anastomoses created with the OCW technique, leading to a narrower anastomotic area, could potentially induce local flow disturbances and, consequently, pose a higher risk for neointimal hyperplasia compared to the CWE technique.

### Limitations

There are some limitations that are important to consider when interpreting the results of the study. While animal models can provide valuable information, they may not always reflect the complex and variable conditions present in human CABG. The small sample size and non-physiological conditions of the study also limit the generalizability of the findings. Additionally, testing with saline instead of blood may affect the results in terms of viscosity. Finally, the gravity pressure used to provide flow is not the same as the natural blood pressure, which may also impact the outcomes. Therefore, further studies with larger sample sizes and more physiological conditions are necessary to confirm the results and evaluate the clinical implications of these findings.

## CONCLUSION

The technique, called CWE, may lead to better hemodynamics by providing a larger anastomosis area and possibly more laminar flow compared to the OCW technique. In addition, the CWE technique may have an advantage in avoiding the purse-string effect, which can occur in the OCW technique. Moreover, the frailty and weakness of coronary arteries, especially in diabetic patients, may increase the risk of anastomosis tearing and leakage, and the CWE technique may be safer in this regard. On the other hand, the OCW technique, where the suture only penetrates the coronary wall, may result in a narrower anastomosis area and create a local flow disturbance, potentially leading to a higher risk of neointimal hyperplasia. However, further studies are needed to evaluate the long-term outcomes and potential risks associated with the CWE technique compared to the OCW technique.

## Abbreviations, Acronyms & Symbols

CABG: Coronary artery bypass grafting
CWE: Coronary wall and epicardial fat tissue
OCW: Only coronary wall
TTFM: Transit-time flow measurement
